# MicroRNAs as Theranostics Targets in Oral Carcinoma Stem Cells

**DOI:** 10.3390/cancers12020340

**Published:** 2020-02-03

**Authors:** Pei-Ling Hsieh, Yi-Wen Liao, Martin Pichler, Cheng-Chia Yu

**Affiliations:** 1 Department of Anatomy, School of Medicine, China Medical University, Taichung 404, Taiwan; plhsieh@mail.cmu.edu.tw; 2 School of Dentistry, Chung Shan Medical University, Taichung 40201, Taiwan; rabbity0225@gmail.com; 3 Department of Dentistry, Chung Shan Medical University Hospital, Taichung 40201, Taiwan; 4 Research Unit of Non-Coding RNAs and Genome Editing, Division of Clinical Oncology, Department of Medicine, Comprehensive Cancer Center Graz, Medical University of Graz, 8036 Graz, Austria; martin.pichler@medunigraz.at; 5 Institute of Oral Sciences, Chung Shan Medical University, Taichung 40201, Taiwan

**Keywords:** head and neck squamous cell carcinoma, oral cancer, cancer stem cells, non-coding RNAs

## Abstract

Oral cancer belongs to head and neck squamous cell carcinoma and has been recognized as one of the most prevalent malignancies worldwide. Recent studies have suggested that cancer stem cells (CSCs) may participate in tumor initiation, metastasis and even recurrence, so the regulation of CSCs has drawn significant attention over the past decade. Among various molecules that are associated with CSCs, non-coding RNAs (ncRNAs) have been indicated as key players in the acquisition and maintenance of cancer stemness. In addition, accumulating studies have shown that the aberrant expression of these ncRNAs may serve as surrogate diagnostic markers or even therapeutic targets for cancer treatment. The current study reviews the previous work by us and others to summarize how these ncRNAs affect oral cancer stemness and their potential theranostic applications. A better understanding of the implication of these ncRNAs in oral tumorigenesis will facilitate the translation of basic ncRNA research into clinical application in the future.

## 1. Introduction

Stem cells are characterized by the capacity for self-renewal and the ability to give rise to differentiated progeny. Cancer stem cells (CSCs) are also considered to possess similar features, which lead to tumor growth, repopulation after treatment and metastasis [1,2]. Indeed, emerging evidence has shown that the existence of CSCs is one of the main obstacles to successful cancer therapy. As such, great effort has been made to target CSCs and eliminate their stemness traits for therapeutic application. In head and neck squamous cell carcinomas (HNSCC), various approaches have been utilized to identify CSCs, such as cluster of differentiation 133 (CD133), cluster of differentiation 133 CD44, aldehyde dehydrogenase 1 (ALDH1) staining or sphere formation [3,4,5]. However, the efficient approach to completely eradicate oral cancer remains lacking. Hence, a better understanding of the mechanism underlying the tumorigenicity and pluripotency of CSCs is still of great interest.

Over the past decades, numerous stem cell factors have been shown to be involved in the regulation of oral CSCs properties via epithelial–mesenchymal transition (EMT), such as octamer-binding transcription factor 4 (Oct4) [5,6,7], Nanog [5,7] or sex determining region Y-box 2 (Sox2) [8]. In accordance with these findings, several EMT mediators have been demonstrated to maintain the cancer stemness characteristics in HNSCC [9,10,11]. For instance, our previous studies have shown that downregulation of S100A4 in oral CSCs reduced their self-renewal and tumorigenic capabilities [10], and co-knockdown of ZEB1 and ZEB2 in HNSCC-CD133^+^ cells inhibited their CSC-like features [11]. Aside from EMT mediators, accumulating studies have revealed that non-coding RNAs also serve as the upstream modulators of CSCs [12,13,14] through various mechanisms, including the aforementioned EMT regulation.

Non-coding RNAs refer to RNA that does not encode a protein and can be divided into numerous categories, including small non-coding RNAs (such as microRNAs; ~19–22 nucleotides in length) and long non-coding RNAs (lncRNAs; more than 200 nucleotides in length) [15]. MicroRNAs (miRs) has been proven to regulate the translational efficiency or stability of targeted mRNAs by interacting with the 3′-untranslated region (3′-UTR) of their targets [16]. Unlike the way miRs post-transcriptionally regulate mRNAs, lncRNAs are implicated in various levels of fundamental epigenetic processes. It has been indicated that lncRNAs could act as molecular signals or as decoys for miR target sites. LncRNAs can also function as a guide to recruit chromatin-modifying enzymes, to target genes or as scaffolds to form ribonucleoprotein complexes [17]. In this review, we summarized the effects of miRs on oral CSC features from our work and others, and sought to broaden our understanding of how they elicit functional outcomes and contribute to the development of effective therapy to oral carcinomas. 

## 2. Let-7 Family

Let-7 was identified as a heterochronic gene in *C. elegans* [18] and one of the first described miR families that act as tumor suppressors. It has been known that there are 12 different let-7 family members expressed in humans [19]. Two key RNA-binding proteins, LIN28A and LIN28B, were shown to inhibit the biogenesis of let-7 families through direct binding to pre-let-7 [20]. It has been previously found that the Lin28B-let-7 pathway positively regulated the expression of stemness factors Oct4 and Sox2, resulting in a reprogramming-like phenomenon. Moreover, it induced a switch of non-CSCs to CSCs with tumor-initiating and self-renewal characteristics in oral CSC [21]. The AT-rich interaction domain molecule 3B (ARID3B) and HMGA2 were revealed as the direct targets of let-7, which could directly regulate the Oct4 and Sox2 promoter activity [21]. Consistently, we demonstrated that overexpression of let-7a led to downregulation of Nanog in ALDH1^+^ HNSCC cells [22]. Besides, we showed that ectopic expression of let-7c or let-7d in oral CSCs repressed the stemness hallmarks and the radio/chemoresistance through suppression of IL-8 or EMT markers, respectively [23,24]. The expression of let-7c and let-7d were indeed lower in lymph node metastatic lesions [23,24], and let-7d expression in HNSCC tumors was proved to be significantly associated with poor survival [25]. Altogether, these findings indicated that the let-7 family function as a suppressor of oral CSCs and a prognostic factor for oral cancer. 

## 3. MicroRNA-200 Family

The miR-200 family consists of two clusters that are located on two different chromosomes, including cluster I (miR-200b, -200a and -429 is located on chromosome 1) and cluster II (miR-200c and -141 is located on chromosome 12) [26]. The role of miR-200 during normal development of salivary glands has been revealed previously. For instance, it has been shown that miR-200c reduced FGFR-dependent epithelial proliferation during submandibular gland morphogenesis through targeting the lipoprotein receptor Vldlr and its ligand reelin [27]. In addition to controlling epithelial morphogenesis during glandular development or regeneration, dysregulation of miR-200 was associated with tumor formation as well. The miR-200 family share common seed sequences, which modulate EMT through direct targeting of ZEB1 or ZEB2 [28]. In fact, it has long been considered that the ZEB/miR-200 feedback loop controls the state of CSCs [29]. In oral squamous cell carcinomas (OSCCs), downregulation of the miR-200 family has been reported [30]. There is a reciprocal correlation between miR-200c/miR-141 and ZEB1 in HNSCC, and overexpression of miR-200c/miR-141 inhibits the migration capacity [31]. The loop of ZEB1/miR-200 has been shown to control the Notch signaling in cancer cells [32], and our previous work demonstrated that miR-200c attenuated tumor growth and metastasis via reducing the expression of BMI1/ZEB1 [33]. BMI1 is an important stem cell regulator, and our finding was consistent with another study showing that miR-200c could directly modulate breast CSCs [34]. We found that the expression levels of miR-200c were significantly downregulated in ALDH1^+^/CD44^+^ HNSCC with increased BMI1 expression. Moreover, we showed that upregulation of miR-200c or knockdown of BMI1 could significantly inhibit the malignant CSC properties, and knockdown of ZEB1 or ZEB2 could increase miR-200c and inhibit BMI1 expression in ALDH1+/CD44+ HNSCC cells, indicating that the interaction among miR-200c, ZEB1/ZEB2 and BMI1 determined the fate of cancer stemness in OSCC. On the other hand, a well-known tumor repressor, p53 was able to bind to the promoter region of miR-200c at multiple sites [35]. As one of the most frequently inactivated tumor suppressor gene in HNSCC [36], loss of p53 expression has been proven to correlate with the metastatic capacity of HNSCC [37]. The p53 mutation may also impair the downstream transcriptional activation of miR-200c, leading to increased CSC characteristics.

## 4. MicroRNA-145

As a putative tumor suppressing miR, miR-145 has been found to be decreased in various cancers and could restrain cancer cell behavior [38,39]. Further evidence to support its tumor suppressive role is that DFF45, an important factor of drug-induced apoptosis, has been shown to be a direct target of miR-145 [40]. It is worth it to mention that the two co-transcribed but distinct miRNAs, miR-143 and miR-145, formed the miR-143/145 cluster and has been intensively studied. Repression of the miR-143/145 cluster was observed in pancreatic cancers after Ras activation, and restoration of these miRNAs abrogated tumorigenesis [41]. Likewise, miR-143 and miR-145 were underexpressed in most HNSCC samples, and loss of miR-143/145 disturbed cellular growth and apoptosis in HNSCCs since the miR-143/145 cluster was able to reduce mouse double minute 2 homolog (MDM2) with increased expression of p21 and BAX [42]. Another study revealed that downregulation of the miR-143/miR-145 cluster resulted in overexpression of activin A in OSCCs, contributing to lymph node metastasis and poor survival [43]. Previously, we have shown that ALDH1^+^CD44^+^ HNSCC cells express reduced levels of miR145, and inhibition of miR-145 was sufficient to drive tumor-initiating features in ALDH1^−^CD44^−^ HNSCC cells [44]. Our data showed that miR-145 was able to directly bind to SOX9 and ADAM17 via their 3’-UTR regions. Moreover, we found that SOX9 directly regulates the ADAM17 promoter and this SOX9/ADAM17 axis determines the miR-145-mediated EMT and CSC characteristics. Additionally, we demonstrated that the miR-145-ADAM17 pathway mediates the secretion of the IL-6 and soluble IL-6 receptor, which may contribute to the maintenance of CSC properties in a paracrine manner. Afterward, we showed that delivery of curcumin attenuates tumor progression in vivo via increasing miR-145 promoter activity [44]. In another study, we revealed that 5-aminolevulinic acid-mediated photodynamic therapy was able to upregulate the expression of miR-145 in oral cancer cells, thereby possessing the potential to reduce the CSC features [45].

Apart from CSC, miR-145 also has been reported to regulate the cancer-associated fibroblasts (CAFs), the most abundant cell types in the tumor microenvironment. It has been shown that upregulation of miR-145 inhibited TGF-β1-induced myofibroblastic transdifferentiation and reverted CAFs towards a normal fibroblast phenotype [46]. CAFs have been indicated to promote stem cell-like properties of hepatocellular carcinoma cells via IL-6/STAT3/Notch signaling [47], and factors secreted by CAFs, such as epidermal growth factor receptor, insulin-like growth factor receptor and platelet-derived growth factor receptor, were proven to enhance stemness in HNSCC [48]. Taken together, these findings suggested that miR-145 affects the CSCs via SOX9/ADAM17 axis to modulate EMT and CSC characteristics, and it also regulates the communication between tumor stroma and CSC through the secretion of paracrine cytokines in order to sustain the cancer stemness hallmarks in HNSCC.

## 5. MicroRNA-21

As one of the most commonly elevated miRNA in a variety of cancers, miR-21 has been known to be located on 17q23.2 within the intron of the TMEM49 gene [49]. Like miR-200, miR-21 also has been shown to regulate the development of salivary glands. It has been reported that the upregulated miR-21 in the mesenchyme enhanced branching morphogenesis in murine submandibular glands. Two target genes of miR-21 (RECK and PDCD4) were downregulated in the mesenchyme, whereas miR-21 expression levels were up-regulated [50]. Aside from modulation of salivary glands, it has been revealed that the expression of miR-21 was consistently increased and associated with severity during oral malignant progression [51]. Several studies have shown that miR-21 is an oncogenic miR that regulates the expression of multiple tumor suppressor genes, such as phosphatase and tensin homolog (PTEN), which was associated with tumor dissemination and clinical outcome [52,53]. It has been shown that inhibition of miR-21 in hepatocellular carcinoma (HCC) cells increased the expression of PTEN, and decreased tumor cell proliferation, migration and invasion [54]. In stem-like populations of HCC cells, a higher expression of miR-21 was discovered, and modulation of miR-21 altered the expression of PTEN [55]. Under hypoxia conditions, miR-21 was found to upregulate PD-L1 expression in the OSCC tumor-derived exosomes via PTEN and regulate the inhibitory role of myeloid-derived suppressor cells [56]. The beneficial effects of integrated exosome inhibition with immune checkpoint inhibition, to target miR-21 and PD-L1 in OSCC-bearing immune-competent mice, also has been demonstrated [56].

Another tumor suppressor, programmed cell death 4 (PDCD4), has been found to be decreased by miR-21 as well. Reis et al. [57] showed that loss of PDCD4 increased the invasive potential of OSCC and downregulation of PDCD4 may be due to the direct binding of miR-21 to the 3′-UTR region of PDCD4 [57]. Consistent with this finding, it has been shown that stimulation of miR-21 expression by hyaluronan/CD44 signaling resulted in a decrease of PDCD4, and upregulation of inhibitors of the apoptosis family of proteins (IAPs) as well as chemoresistance [58]. Their data suggested that the activation of Nanog/STAT3 signaling participated in this miR-21 production. In conformity with this result, it has been shown that inhibition of nuclear translocation of phosphorylated STAT3 abrogated the downstream miR-21/β-catenin axis, leading to suppression of tumor growth and invasion in HNSCC [59].

In addition to modulating the tumor suppressor genes, miR-21 can affect the CSC properties. Our previous work has demonstrated that downregulation of miR-21 in OSCC-CSCs by berberine reduced ALDH1 activity and inhibited self-renewal, migration and invasion capabilities [60]. Moreover, it has been reported that miR-21 negatively regulated miR-145 via the Ras signaling pathway in colon CSCs [61]. Since we have shown that miR-145 inhibited OSCC-CSCs via targeting the Sox9/ADAM17 axis [44], it is worthy of further exploration to reveal the network between miR-21 and miR-145 and whether their interaction plays a critical role in the regulation of OSCC-CSCs.

## 6. MicroRNA-1

MiR-1 is coded by two different precursors of miR-1-1 and miR-1-2, and functions as a tumor suppressor in various types of cancers [62,63]. It is known that miR-1-1/miR-133a-2 and miR-1-2/miR-133a-1 are clustered on different chromosomal regions in the human genome, 20q13.33 and 18q11.2, respectively. Hsa-miR-133a-1 and hsa-miR-1-2 both have been found to be associated with overall survival in OSCC [64]. In OSCC cells with ectopic miR-1 expression, a higher proportion of apoptosis and G0/G1 arrest have been observed. They also proved that restoration of miR-1 in cancer cells inhibited cell proliferation, invasion and migration via direct regulation of TAGLN2 by miR-1 [63]. Another study revealed that miR-1-3p inhibited the proliferation and migration of OSCCs by targeting DKK1, a potent inhibitor of the Wnt signaling pathway [65]. Besides, miR-1 was found to regulate two receptors, epidermal growth factor receptor (EGFR) and hepatocyte growth factor receptor (c-MET) in HNSCC [66]. In an experimental prostate adenocarcinoma model, it has been shown that Slug was a direct target of miR-1 and miR-200 transcription, which acted in a self-reinforcing regulatory loop to amplify EMT [67]. They showed that overexpression of miR-1 or miR-200 inhibited EMT and tumorigenesis. In line with this previous work, we found similar results and showed that miR-1 expression was downregulated in oral cancer. Our results demonstrated that overexpression of miR-1 inhibited CSC properties and was able to reduce Slug and increase E-cadherin [68]. Collectively, the current findings support that miR-1 play a tumor suppressive role in OSCC via direct binding to TAGLN2 or Slug, as well as its role in modulation of the Wnt, EGFR or c-MET pathways.

## 7. MicroRNA-204

A recent systematic review and meta-analysis has revealed that downregulation of miR-204 was correlated with poor prognosis in OSCC [69]. In other cancer types, it appears miR-204 also serves as a tumor suppressor. For instance, it has been shown that miR-204 inhibited cell proliferation, migration and invasion by targeting PCNA-1 in lung cancer cells [70] or by targeting ZEB2 in hepatocellular cancer cells [71]. Several reports have suggested that a number of lncRNAs promoted cancer progression via sponge miR-204 [72,73]. In the regulation of cancer stemness, it has been demonstrated that loss of miR-204 expression enhanced CSC phenotypes through targeting Sox4 and the migration-promoting receptor EphB2 in glioma cells [74]. MiR-204 has also been shown to reverse drug resistance and ameliorate CSC phenotypes by degrading FAP-α in glioblastoma [75].

As for oral cancer, it has been previously shown that cell proliferation marker Ki67 and cell cycle protein cyclin D1 were both down-regulated in OSCC cells overexpressed with miR-204-5p. In addition to cell growth, miR-204-5p also affected the invasiveness of the cells, possibly by direct regulation of CXCR4 [76]. Consistently, our earlier report found that the expression level of miR-204 in OSCC-CSCs was lower, and restoration of miR-204 suppressed cancer stemness and in vivo tumor-growth. Our finding indicated that miR-204 was able to bind to the 3′-UTR-regions of Slug and Sox4 and suppress their expression in OSCC-CSCs. Moreover, oral feeding of EGCG effectively alleviated tumor-progression via upregulation of miR-204 [77]. In view of these reports together with our previous observation, miR-204 is an important mediator to regulate the cancer stemness via binding to Sox4 and FAP-α.

## 8. MicroRNA-218

There are two genes encoding miR-218, miR-218-1 and miR-218-2, which are located at 4p15.31 and 5q35.1 within the introns of Slit2 and Slit3, respectively. In brain cancer, it is well known that miR-218 regulates cell migration/invasion, proliferation and apoptosis by targeting different genes [78]. For instance, miR-218 inhibited cell aggressiveness and cancer stemness features in glioma by targeting BMI1 [79]. The effects of BMI1 on stemness are thought to be related to the upregulation of p21^CIP1^ and with decreased Akt activation [80]. In addition, it has been revealed that miR-218 negatively regulated the IL-6 receptor and JAK3 gene expression in lung cancer cells by directly targeting their 3′-UTR regions, thereby repressing tumor sphere formation and other tumorigenesis features [81]. Another study showed that administration of miR-218-mimic downregulated the expression of Slug and ZEB2 in lung cancer cells [82], but the detailed mechanism still remains unclear. In OSCC, HPV E6-mediated paxillin expression has been shown to promote colony formation and invasion in oral cancer via decreasing miR-218 [83]. We also conducted a study to elucidate the function of miR-218 in OSCC-CSCs and examined the anti-OSCC effect of a natural compound, andrographolide, by targeting miR-218. We showed that overexpression of miR-218 significantly suppressed the radio-resistance, self-renewal and invasion abilities in OSCC-CSCs. By demonstration of silencing the endogenous miR-218-induced sphere-forming capability in non-CSCs, and that knockdown of Bmi1 abolished this phenomenon, we were able to prove that miR-218-induced downregulation of Bmi1 diminished stemness properties in OSCC [84]. Nevertheless, a later study showed that ectopic expression of miR-218 induced cell survival and resistance to cisplatin by direct inhibition of PPP2R5A, a repressor of Wnt signaling, and promoted miR-218-mediated Wnt activation in oral cancer [85]. Since one miRNA may regulate multiple mRNAs due to the allowance of a mismatched base pairing with mRNAs, further investigation is required to elucidate whether the regulation of miR-218 in oral CSCs is different from cisplatin-resistant cancer cells.

## 9. MicroRNA-494

MiR-494 was initially found to be highly expressed in retinoblastoma [86], and its function in carcinogenesis as a tumor suppressor or oncogenic factor varies according to the cancer type. MiR-494 has been revealed to possess a global regulatory role in cell cycle progression to induce G_2_/M arrest in human cholangiocarcinoma [87]. Downregulation of miR-494 has been found in pancreatic ductal adenocarcinoma, and Li et al. [88] showed that miR-494 was able to serve as a negative regulator of FOXM1 and affect nuclear localization of β-catenin [88]. MiR-494 has been shown to suppress cell proliferation in lung cancer A549 cells through binding to insulin-like growth factor 2-binding protein 1 (IGF2BP1) and affecting its downstream target, IGF2 [89]. A later study showed that lncRNA ATB downregulated miR-494 to promote A549 cell proliferation via Akt and the JAK/STAT3 pathway [90]. However, the expression of miR-494 was found to be significantly higher in the tissues of non-small cell lung cancer, and the overexpression of miR-494 and lower expression of PTEN were positively correlated with TNM staging and lymph node metastasis [91]. In ovarian carcinomas, the expression of miR-494 was decreased and IGF1R also has been identified as a direct target of miR-494 [92]. They demonstrated that overexpression of miR-494 obviously decreased p-Akt and p-ERK expression. MiR-494 inhibited ovarian cancer cell proliferation by targeting FGFR2 and inducing apoptosis as well [93]. 

On the other hand, it has been reported that miR-494 promoted liver and cervical cell proliferation, migration and drug resistance by targeting PTEN [94,95]. MiR-494 was overexpressed in human hepatocellular carcinoma cells (HCC) and able to regulate the G1/S cell cycle transition through targeting of the tumor suppressor Mutated in Colorectal Cancer [96]. Moreover, miR-494 could trigger gene silencing of numerous invasion-suppressor miRNAs by inhibiting genomic DNA demethylation, resulting in tumor vascular invasion [97]. As for cancer stemness, miR-494-3p was proven to target BMI1 and inhibit various features of cancer stemness in breast cancer. In addition, miR-494-3p expression was inversely correlated with patient survival [98]. MiR-494 also has been shown to influence the stemness properties of HCC by increasing various stemness genes, such as PROM1, Oct4 and Sox2, as well as ABCG2 transporter levels [99]. In oral cancer, miR-494 was shown to be the most significantly underexpressed miRNAs, and it repressed the expression of HOXA10 and reduced the proliferation of oral cancer cells [100]. Our previous work showed that upregulation of miR-494 by silibinin inhibited ALDH1 activity, CD133 positivity and other stemness signatures in ALDH1+CD44+ oral cancer cells. We demonstrated that activation of miR-494-inhibited both Bmi1 and ADAM10 expression in OSCC-CSCs [101], where miR-494-3p could enhance the radiosensitivity and induce a senescence pathway in oral cancer cells [102]. These results indicated that upregulation of miR-494 may exert an anti-cancer effect in oral cancer.

## 10. MicroRNA-1246

MiR-1246 was identified as a target of the p53 family and could directly inhibit the expression of Down syndrome-associated DYRK1A, therefore activating NFAT1c and inducing apoptosis [103]. The p53-induced miR-1246 also inhibited the cell growth of HCC through targeting the oncogene nuclear factor I/B (NFIB) [104]. Another study showed that miR-1246 enhanced migration and invasion of HCC via down-regulation of Cell adhesion molecule 1 (CADM1) [105]. In esophageal squamous cell carcinoma, serum miR-1246 has strong potential as a novel diagnostic and prognostic biomarker [106]. The higher expression of miR-1246 in OSCC tissues and oral cancer cell lines were correlated with TNM stage and patients’ survival [107]. In fact, exosomal miR-1246 has been demonstrated to increase cell motility by directly targeting DENN/MADD Domain Containing 2D (DENND2D) in OSCC [108]. In terms of cancer stemness, miR-1246 was shown to increase tumor-initiating potential and induced drug resistance in pancreatic cancer via CCNG2 [109]. Oct4 was found to regulate the expression of miR-1246 and drive β-catenin activation in liver CSCs. Chai et al. [110] demonstrated that miR-1246 promoted cancer stemness by activation of the Wnt/β-catenin pathway through suppressing AXIN2 and glycogen synthase kinase 3β (GSK3β) [110]. The upregulated miR-1246 in the lung CD166+ CSC contributed to tumorigenesis and was crucial to the metastatic ability of CSCs via targeting MT1G [111]. In agreement with the previous work, we found that the expression level of miR-1246 in OSCC tissues was upregulated, which was correlated with poor survival. We showed that there was a positive relationship between miR-1246 and stemness markers, and miR-1246 promoted cancer stemness via suppression of CCNG2 [112]. Our results demonstrated that inhibition of miR-1246 resulted in the higher sensitization of cisplatin, suggesting that miR-1246 could function as a suitable target to ameliorate the aggressiveness of OSCC. Collectively, these studies indicated that the aberrant expression of miR-1246 could serve as a prognosis biomarker in various cancers, including OSCC. MiR-1246 modulated the cancer stemness in multiple cancers and we showed that miR-1246 was a good theranostic target in OSCC. The aforementioned miRs are listed in Table 1.

## 11. Conclusions

**Figure 1:**
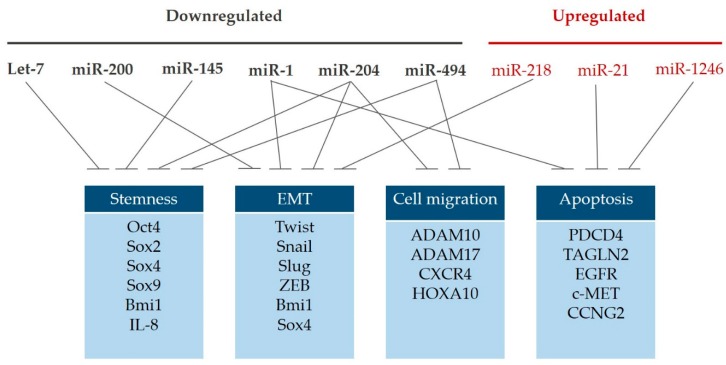
These aberrantly expressed miRs concertedly regulate key properties that affect cancer stemness in OSCC, especially cancer stemness and epithelial–mesenchymal transition (EMT).

MicroRNAs can act not only as oncogenes but also as tumor suppressors. In this review, we summarized our previous work regarding the function of miRs in the regulation of OSCC-CSC and related these findings with other studies (Figure 1). We also showed the effect of modulation of these miRs on suppression of cancer aggressiveness. Hopefully, these results will provide insight into targeting miRs as novel strategies to eradicate OSCC-CSC.

## Figures and Tables

**Table 1:** The expression of non-coding RNAs in oral squamous cell carcinoma (OSCC) tissues, OSCC cell lines or cancer stem cells.

Non-Coding RNA	Expression	Molecular Target (s)/Associated Factor (s)	Note	Reference
Let-7	Downregulation	ARID3B and HMGA2	Lin28B-let-7 pathway positively regulates the expression of stemness factors, Oct4 and Sox2	Chien et al. [21]
Let-7a	Downregulated in cancer tissues; lower in lymph node metastatic lesions		Let-7a or Nanog treatment effectively reduced the number of lung metastases and tumour size in vivo	Yu et al. [22]
Let-7c	Downregulation	IL-8	Let-7c downregulated the stemness hallmarks and the radio/chemoresistance	Peng et al. [23]
Let-7d	Downregulation	Twist and Snail	Let-7d negatively modulates EMT	Chang et al. [24]
Let-7d	Downregulation		Hsa-let-7d expression are significantly associated with poor prognosis	Childs et al. [25]
miR-200 family	Downregulation	ZEB	No significant associations were observed between expression levels of miR-200 family, tumor grade and nodal stage of tumor samples	Brabletz et al. [29], and Arunkumar et al. [30]
miR-200c	Downregulated in cancer tissues; lower in lymph node metastatic lesions	Bmi1	Overexpression of miR-200c reduces stemness, EMT and sensitizes ALDH1+CD44+ HNSCCs to radio- and chemotherapy	Lo et al. [33]
miR-145	Downregulation	MDM2	Ectopic expression of miR-143 and miR-145 leads to inhibition of MDM2 and the upregulation of p53. Overexpression of p53 led to increased miR-143 and miR-145.	Zhang et al. [42]
miR-145		INHBA	Overexpression of activin A is correlated with lymph node metastasis and overall survival, which is associated with the downregulation of miR-143/miR-145 cluster	Bufalino et al. [43]
miR-145	Downregulation	SOX9 and ADAM17	miR-145 targets the SOX9/ADAM17 axis to suppress a IL-6-mediated paracrine signaling to regulate CSC properties	Yu et al. [44]
miR-21	Overexpression	PDCD4	PDCD4 under-expression in OSCC is associated with poor prognosis and regulated by miR-21	Reis et al. [57]
miR-21	Overexpression	PDCD4 and IAP	Stimulation of miR-21 expression by HA/CD44 signaling is Nanog/Stat-3-dependent in HNSCC cells, leading to PDCD4 reduction, IAP upregulation and chemoresistance	Bourguignon et al. [58]
miR-21	Overexpression		Berberine reduces miR-21, leading to the reduced characteristics of CSCs	Lin et al. [60]
miR-1	Downregulation	TAGLN2	miR-1 suppresses cell proliferation, migration and invasion and promotes apoptosis and cell cycle arrest	Nohata et al. [63]
miR-1	Downregulation	EGFR and c-MET	miR-1 and miR-206 inhibit EGFR and c-MET downstream signal cascades	Koshizuka et al. [66]
miR-1	Downregulated in cancer tissues; lower in metastatic lesions	Slug	miR-1 decreases cancer stem cells properties	Peng et al. [68]
MiR-1-3p	Downregulation	DKK1	miR-1-3p suppresses OSCC cell proliferation, migration, and invasiveness	Wang et al. [65]
miR-204-5p	Downregulation	CXCR4	miR-204-5p may function as an inhibitory miR in OSCC by targeting CXCR4	Wang et al. [76]
miR-204	Downregulation	Slug and Sox4	miR-204-mediated suppression of cancer stemness and EMT properties by targeting Slug and Sox4	Yu et al. [77]
miR-218		Bmi1	Andrographolide increases the expression of miR-218, which suppresses cancer stemness and invasiveness by targeting Bmi1	Yang et al. [81]
miR-218	Overexpression	PPP2R5A	Suppression of miR-218 causes apoptosis and enhances sensitivity to cisplatin. PPP2R5A overexpression or β-catenin knockdown inhibits miR-218-mediated Wnt activation	Zhuang et al. [85]
miR-494	Downregulation	HOXA10	miR-494 represses the expression of HOXA10 and reduces the proliferation of oral cancer cells	Libório-Kimura et al. [100]
miR-494		Bmi1 and ADAM10	knockdown of miR-494 in non-CSCs enhances cancer stemness and oncogenicity, while co-knockdown of Bmi1 and ADAM10 effectively reverses it	Chang et al. [101]
miR-494-3p		Bmi1	miR-494-3p enhances radiosensitivity and induces cellular senescence	Weng et al. [102]
miR-1246	Overexpression		High miR-1246 expression is associated with poor prognosis	Liao et al. [103]
miR-1246		DENND2D	Exosomal miR-1246 induces cell motility and invasion through the regulation of DENND2D	Sakha et al. [108]
miR-1246	Overexpression	CCNG2	Higher expression of miR-1246 is associated with poor prognosis and miR-1246-inhibited CCNG2 contributes to the cancer stemness	Lin et al. [112]
